# COMMENTARY ON “EFFECTS OF UPPER LIMB VIBRATORY STIMULATION TRAINING ON MOTOR SYMPTOMS IN PARKINSON’S DISEASE: AN OBSERVATIONAL STUDY”

**DOI:** 10.2340/jrm.v56.40920

**Published:** 2024-07-29

**Authors:** Mebanpynjop DOHTDONG, Shanika SHARMA, Varun KALIA

**Affiliations:** Department of Physiotherapy, Lovely School of Allied Medical Sciences, Lovely Professional University, Phagwara, Punjab, India

We read with great interest the article by Varalta et al. (1). The authors did an impressive job of delving into the problems faced by Parkinson’s patients, as tremor is a difficult PD symptom that greatly impairs manual abilities and daily activities. However, we are writing to share our concerns regarding this recent study published in the Journal of Rehabilitation Medicine. While the study shows interesting findings concerning the potential benefits of vibratory stimulation training for tremor reduction and motor functionality in Parkinson’s disease (PD) patients, several drawbacks should be addressed to ensure a thorough understanding of the intervention’s efficacy.

First, there is a mismatch between the title and the methodology section of the study. The paper’s title highlights the vibratory machine’s impact on tremor and upper limb motor functions, yet the study also assesses cognitive function, as found in the methodology section. As a result, the title should be updated to reflect this broader scope.

Additionally, the study’s title indicates that it is an observational study, meaning no active intervention was given by the researcher and merely observing natural outcomes without manipulating any variable. In contrast, the methodology section of the study indicates an intervention (upper limb vibratory stimulation training) was applied to the participants, and outcomes were measured before and after the intervention. This study design is consistent with that of a clinical trial (experimental study). In an observational study, individuals are monitored without changing the study setting or the subjects themselves. Researchers watch and gather data on people’s features and results without intervening. However, a clinical trial is a research study that evaluates the efficacy and safety of medicinal, surgical, or behavioural therapies on a group of participants and measurements are taken before and after the intervention to assess its effects (2). Moreover, as no control group is present in this study, and only one group is receiving an intervention, the design of this study can be considered as a single-arm uncontrolled pre–post study design (3).

Second, there is a mismatch between the introduction and methodology of the study. The introduction does not include the background of cognitive impairment in Parkinson’s disease, and even the objective of the study is only to evaluate the effects of an upper limb (UL) vibratory rehabilitation programme using a specific device (Armshake^®^, Move It GmbH, Bochum, Germany) on tremor and motor functionality in patients with PD but the methodology section has incorporated a scale for assessing cognitive dysfunction as well. It is necessary to establish and explain the importance of testing cognitive function in Parkinson’s disease. Furthermore, the paper should also explain why there is a possibility of changes in cognitive performance simply by giving treatment for improving upper limb motor functions of Parkinson’s disease.

Furthermore, the study has made no mention of the sampling procedure. While such studies do not include randomization into separate groups, precise sampling procedures must be used to guarantee that the research population is suitable and representative of the larger patient population for which the intervention is designed (4).

Additionally, neither the reliability nor the validity of the outcome measures used are mentioned. This is a significant omission since these measures ensure that the tools employed are both reliable and valid (5). This information is routinely included in clinical studies to increase the reliability and robustness of the findings.

Lastly, the study fails to effectively address potential confounding variables such as treatment status or disease severity, which may impact treatment outcomes. Including these characteristics as covariates in the analysis, or stratifying the sample by disease stage, would improve the study’s rigour.

Addressing the mentioned drawbacks, such as revising the title, explaining the relationship between motor and cognitive functions, reporting the reliability and validity of outcome measures, providing background on cognitive dysfunction, and detailing the sampling methodology, would improve the study’s validity. This would help us gain a better grasp of the effectiveness of vibratory stimulation training in treating motor symptoms in PD patients.
