# Supplementary material for: COMMENTARY ON “EFFECTS OF UPPER LIMB VIBRATORY STIMULATION TRAINING ON MOTOR SYMPTOMS IN PARKINSON’S DISEASE: AN OBSERVATIONAL STUDY”
Source: J Rehabil Med. 2024 Jul 29;56:40920. doi: 10.2340/jrm.v56.40920 (PMC11302131; doi:10.2340/jrm.v56.40920)
Supplement: Related texts

# Reply: RESPONSE TO THE COMMENTARY ON “EFFECTS OF UPPER LIMB VIBRATORY STIMULATION TRAINING ON MOTOR SYMPTOMS IN PARKINSON’S DISEASE: AN OBSERVATIONAL STUDY”

**Authors:** Alessandro PICELLI, Nicola SMANIA, Valentina VARALTA

**Affiliations:** 1 Neuromotor and Cognitive Rehabilitation Research Center, Department of Neurosciences, Biomedicine and Movement Sciences, University of Verona, Verona, Italy; 2 Neurorehabilitation Unit, Department of Neurosciences, University Hospital of Verona, Verona, Italy

First of all, we would like to thank Mebanpynjop Dohtdong and colleagues for their interest in and comments on our recent article (1).

Tremor is a cardinal motor symptom of Parkinson’s disease (PD), affecting up to 80% of patients (2). This condition significantly impacts the quality of life, leading to disability and considerable limitations in activities of daily living (3). The Armshake^®^ device (Move It GmbH, Bochum, Germany) is marketed in Europe specifically for treating tremor and its sequelae in patients with PD. As the Armshake^®^ is currently in use at our clinical unit, we decided to include it in some research tasks. These activities were limited to the intended purposes specified by the manufacturer (“in-label” use) and did not include any additional interventions beyond those performed under normal conditions of device use (for clarity, we did not impose any experimental or controlled conditions but simply collected data from patients undergoing normal clinical practice). In this context, our investigation concerning the effects of Armshake^®^ should be considered a prospective observational study (1, 4).

Motor behaviour results from a complex integration between cortical and subcortical areas, encompassing the motor, cognitive, and motivational aspects of movement (5). This close interplay (mainly based on dopaminergic projections) enables the learning, control, and expression of habitual (automatic) actions, which become dysfunctional in PD (6). Neuromotor approaches have been found to improve motor and cognitive performance in patients with PD (7–9). Therefore, we collected data on the motor and cognitive effects of the Armshake® device during our observational research activity. Considering the large number of outcome measures recorded from a sample of 20 patients with PD, we decided to present our results in 2 papers. The first deals with the effects of the Armshake® device treatment on cognition and was published in 2022 in Brain Sciences (4). This is the reason why our article recently published in the Journal of Rehabilitation Medicine (JRM) reports predominantly on the motor effects of vibratory stimulation training performed with the Armshake® (1).

In our study, we evaluated patients at 3 timepoints (i.e. before treatment, at the end of treatment, and 30 days after the end of treatment) according to some primary (i.e. the Fahn-Tolosa-Marin Tremor Rating Scale [FTMTRS]) and secondary (i.e. the Unified Parkinson’s Disease Rating Scale [UPDRS]; the Purdue Pegboard test [PPT]; the Disability of the Arm, Shoulder and Hand [DASH] questionnaire; the Montreal Cognitive Assessment [MoCA]) outcomes. With regard to the reliability/validity of our outcome measures, the FTMTRS is recommended by the Movement Disorder Society to assess tremor severity because of its good overall clinimetric properties (i.e., reliability, validity, and sensitivity to change) (10). The UPDRS – part III is reported to have excellent validity and internal consistency as well as adequate ceiling effects (11). The PPT showed high test–retest reliability for measuring hand dexterity in PD. It has intraclass correlation coefficients (ICCs) ≥ 0.90 and very good interrater reliability (ICCs > 0.99) (12). The DASH Italian version has good test–retest reliability (function/symptoms: ICCs = 0.89; sport/music ICCs = 0.75; work: ICCs = 0.84) (13). The MoCA is validated for the screening of mild cognitive dysfunction in PD. It has excellent test–retest (ICCs = 0.79) and interrater reliability (ICCs = 0.81) (14).

As to enrolment of the study population, we applied a consecutive sampling procedure. All patients were evaluated and treated during the “ON phase” of medication (i.e., between 1 and 2.5 h after last intake). The disease stage was defined according to the Hoehn and Yahr (H and Y) scale (15). In order to address the concern regarding potential confounders, a one-way ANCOVA was conducted to determine whether disease severity might impact the primary outcome. We confirm the significant differences found on the FTMTRS between timepoints (F = 4.83; *p* = 0.045) after controlling for the H&Y stage.

In conclusion, we agree with Mebanpynjop Dohtdong and colleagues concerning the importance of motor-cognitive interaction mechanisms, which open to some intriguing possibilities for PD rehabilitation. From this perspective, the results of our study on 20 patients with PD treated with the Armshake® device, published in Brain Sciences and JRM, should be considered, and read, as one (1, 4).

## References

[1] Varalta V, Righetti A, Evangelista E, Vantini A, Martoni A, Tamburin S, et al. Effects of upper limb vibratory stimulation training on motor symptoms in Parkinson’s disease: an observational study. J Rehabil Med 2024; 56: jrm19495. DOI: 10.2340/jrm.v56.19495PMC1091097738407431

[2] Bloem BR, Okun MS, Klein C. Parkinson’s disease. Lancet 2021; 12: 2284–2303. DOI: 10.1016/S0140-6736(21)00218-X33848468

[3] Quinn L, Busse M, Dal Bello-Haas V. Management of upper extremity dysfunction in people with Parkinson disease and Huntington disease: facilitating outcomes across the disease lifespan. J Hand Ther 2013; 26: 148–155. DOI:10.1016/j.jht.2012.11.00123231827

[4] Varalta V, Evangelista E, Righetti A, Morone G, Tamburin S, Picelli A, et al. Effect of upper limb motor rehabilitation on cognition in Parkinson’s disease: an observational study. Brain Sci 2022; 12: 1684. DOI: 10.3390/brainsci1212168436552144 PMC9775162

[5] Ferrazzoli D, Ortelli P, Madeo G, Giladi N, Petzinger GM, Frazzitta G. Basal ganglia and beyond: the interplay between motor and cognitive aspects in Parkinson’s disease rehabilitation. Neurosci Biobehav Rev 2018; 90: 294–308. DOI: 10.1016/j.neubiorev.2018.05.00729733882

[6] Nieuwboer A, Rochester L, Müncks L, Swinnen SP. Motor learning in Parkinson’s disease: limitations and potential for rehabilitation. Parkinsonism Relat Disord 2009; 15: S53–88. DOI: 10.1016/S1353-8020(09)70781-320083008

[7] Varalta V, Picelli A, Fonte C, Amato S, Melotti C, Zatezalo V, et al. Relationship between cognitive performance and motor dysfunction in patients with Parkinson’s disease: a pilot cross-sectional study. Biomed Res Int 2015; 2015: 365959. DOI: 10.1155/2015/36595925918713 PMC4396143

[8] Picelli A, Varalta V, Melotti C, Zatezalo V, Fonte C, Amato S, et al. Effects of treadmill training on cognitive and motor features of patients with mild to moderate Parkinson’s disease: a pilot, single-blind, randomized controlled trial. Funct Neurol 2016; 31: 25–31. DOI: 10.11138/fneur/2016.31.1.02527027891 PMC4819815

[9] Varalta V, Poiese P, Recchia S, Montagnana B, Fonte C, Filippetti M, et al. Physiotherapy versus consecutive physiotherapy and cognitive treatment in people with Parkinson’s disease: a pilot randomized cross-over study. J Pers Med 2021; 11: 687. DOI: 10.3390/jpm1108068734442331 PMC8399749

[10] Elble R, Bain P, Forjaz MJ, Haubenberger D, Testa C, Goetz CG, et al. Task force report: scales for screening and evaluating tremor: critique and recommendations. Mov Disord 2013; 28: 1793–800. DOI: 10.1002/mds.2564824038576

[11] Goetz CG, Tilley BC, Shaftman SR, Stebbins GT, Fahn S, Martinez-Martin P, et al. Movement Disorder Society-sponsored revision of the Unified Parkinson’s Disease Rating Scale (MDS-UPDRS): scale presentation and clinimetric testing results. Mov Disord 2008; 23: 2129–2170. DOI: 10.1002/mds.22340.19025984

[12] Proud EL, Bilney B, Miller KJ, Morris ME, McGinley JL. Measuring hand dexterity in people with Parkinson’s disease: reliability of pegboard tests. Am J Occup Ther 2019; 73: 7304205050p1-7304205050p8. DOI: 10.5014/ajot.2019.03111231318669

[13] Padua R, Padua L, Ceccarelli E, Romanini E, Zanoli G, Amadio PC, et al. Italian version of the Disability of the Arm, Shoulder and Hand (DASH) questionnaire: cross-cultural adaptation and validation. J Hand Surg Br 2003; 28: 179–186. DOI: 10.1016/s0266-7681(02)00303-012631494

[14] Gill DJ, Freshman A, Blender JA, Ravina B. The Montreal cognitive assessment as a screening tool for cognitive impairment in Parkinson’s disease. Mov Disord 2008; 23: 1043–1046. DOI: 10.1002/mds.2201718381646

[15] Goetz CG, Poewe W, Rascol O, Sampaio C, Stebbins GT, Counsell C, et al. Movement Disorder Society Task Force report on the Hoehn and Yahr staging scale: status and recommendations. Mov Disord 2004; 19: 1020–1028. DOI: 10.1002/mds.2021315372591
